# Improving reporting of observational studies of interventions: The TARGET guideline

**DOI:** 10.1371/journal.pmed.1004787

**Published:** 2025-10-15

**Authors:** Harrison J. Hansford, James H. McAuley, Aidan G. Cashin

**Affiliations:** 1 School of Health Sciences, Faculty of Medicine and Health, University of New South Wales, Sydney, New South Wales, Australia; 2 Centre for Pain IMPACT, Neuroscience Research Australia, Sydney, New South Wales, Australia

## Abstract

In this Perspective, Harrison Hansford and colleagues outline how the TARGET guidelines can improve the reporting transparency of observational studies emulating a target trial and help readers appraise and apply the results.

## Teaser

Emulating a target trial when conducting an observational study of interventions can reduce the likelihood of design-related biases. The TARGET guideline aims to improve the reporting transparency of observational studies emulating a target trial and help readers appraise and apply the results.

## Introduction

Improved availability and access to routinely collected data have increased focus on the role of observational data to assess the benefits and harms of interventions. However, analyses of observational data are often subject to biases, some inadvertently introduced by the investigators (e.g., selection bias due to immortal time) and some that are inherent in the non-randomized nature of the data (e.g., confounding). Biases that are introduced by the investigators are often termed “design-related biases” [1]. These biases are common in observational analyses. In a representative sample of 200 observational studies of drug interventions published in 2022, 70% of studies had at least one design-related bias [2]. Investigators can avoid these design-related biases when they align their observational study design with that of a randomized trial, an approach which has become known as target trial emulation (TTE) [3].

## The target trial framework

TTE is a methodological framework to support causal inference from analyses of observational data [3]. The framework has two components: (i) Specifying the target trial and (ii) emulating the target trial with the observational data. Specification of the target trial involves outlining all relevant protocol components of the hypothetical randomized trial (the target trial) that would be conducted to answer the clinical question; this is the causal estimand. Then, the observational emulation involves mapping (emulating) the observational data to the target trial. The target trial is usually designed iteratively based on the information available in the observational data so that it can be emulated closely, given the data constraints. An appropriately specified target trial that is closely emulated with the data should avoid design-related biases, allowing investigators to focus on challenges due to data quality and confounding [3].

Although randomized trials remain the preferred design for evaluating the comparative effects of interventions, TTEs can be used to inform decision-making when such trials are infeasible, unethical, or take too long. This includes settings such as under-resourced low-middle income settings or populations such as pregnant women, where trials may be difficult or not possible, yet relevant evidence is required [4,5]. Due to the increasing availability of data, TTEs can often be conducted rapidly to inform decision-making when trial evidence is not available. For example, following the initial randomized trials demonstrating the efficacy of COVID-19 vaccines compared to placebo, many decision-makers wanted to know which vaccine was more effective and safer compared to another. However, large, comparative randomized trials were unlikely to be conducted by drug manufacturers, and instead, TTEs using routinely collected data demonstrated modest benefits of the BNT162b2 vaccine compared to mRNA-1273 [6].

In settings where randomized trial data are available, TTEs can also be used to extend findings by first benchmarking observational analyses against the published trial findings, before extending the analysis in observational data to broader populations, timepoints, or comparisons [7,8]. This process extends the utility of the randomized trial and can help increase trust in the observational analysis.

Due to increased availability of routinely collected “real-world” data, regulatory bodies worldwide are incorporating observational studies of interventions into their decision-making [9]. The target trial framework is endorsed by the UK National Institute of Health and Care Excellence and the European Medicines Agency to maximize the value of observational studies of interventions. TTEs may prove beneficial for decision-making regarding the comparative benefits and harms and post-marketing surveillance of approved interventions by reducing the risk of design-related biases.

For TTEs to inform decision-making in any context, the target trial protocol, and how it was emulated must be transparently reported. In practice, however, these critical components are poorly reported [10]. To assist authors when designing and reporting their TTEs, we developed the TrAnsparent ReportinG of studies Emulating a Target trial (TARGET) guideline [1,11,12].

## The role of the TARGET guideline

The TARGET guideline was developed as a standalone reporting guideline recommending a minimum set of items to be reported, in the form of a 21-item checklist [1]. It provides guidance for reporting observational studies of interventions explicitly emulating a parallel group, individually randomized target trial, with adjustment for baseline confounders. Complete and transparent reporting of the TARGET checklist items should facilitate readers’ critical appraisal and interpretation of TTEs. The TARGET guideline may also, although not the explicit intention, help improve the methods and conduct of observational analyses of interventions. By serving as an educational resource, it helps clarify key issues and considerations that should be addressed.

The checklist, relevant reporting templates, and resources are freely available on the TARGET website (https://target-guideline.org) to assist investigators in using the guideline. Detailed information on the rationale for and interpretation of each item, including examples of reporting, will be provided in a separate “explanation and elaboration” document, and non-English translated checklists are currently in preparation. Whilst adherence to TARGET may result in more complete and consistent reporting, there will likely be challenges with its uptake, commonly seen across health and medical reporting guidelines [13]. Common barriers include lack of awareness, unclear benefits of use, and the increased burden required on the researcher [14].

There are several strategies that may improve the awareness of investigators to the TARGET guideline, which could improve the quality of observational studies of interventions. Journals and publishers could recommend (or ideally, mandate) authors to submit a completed TARGET checklist alongside submissions of observational studies of interventions. Having a completed checklist at submission may improve reporting quality, which facilitates initial appraisal for critical flaws, speeding up the editorial and peer-review processes. Peer reviewers may also be able to use TARGET as a tool to check whether key aspects of TTEs are reported, and can encourage authors to use it when revising their article.

## Conclusions

Appropriate application of the target trial framework can eliminate design-related biases and improve trust in the increasing number of published studies investigating the comparative effectiveness of interventions with observational data. Complete and transparent reporting will maximize the benefits from these studies. The TARGET guideline provides recommendations to support authors to completely and transparently report their observational studies of interventions. Application of these recommendations may improve the transparency of reporting and strengthen peer review, as well as helping researchers, clinicians, and other stakeholders interpret and apply the study findings.

## Abbreviations

TARGET: TrAnsparent ReportinG of studies Emulating a Target trial
TTE: target trial emulation
